# Exercise Improves Cognitive Function—A Randomized Trial on the Effects of Physical Activity on Cognition in Type 2 Diabetes Patients

**DOI:** 10.3390/jpm11060530

**Published:** 2021-06-09

**Authors:** Roman Leischik, Katharina Schwarz, Patrick Bank, Ania Brzek, Birgit Dworrak, Markus Strauss, Henning Litwitz, Christian Erik Gerlach

**Affiliations:** 1 Department of Cardiology, Prevention and Sports Medicine, Faculty of Health, School of Medicine, University Witten/Herdecke, 58095 Hagen, Germany; katharina.schwarz@pinshof.de (K.S.); patrick.bank@rub.de (P.B.); B.Dworrak@gmx.de (B.D.); markus.strauss@uni-wh.de (M.S.); h.littwitz@gmx.de (H.L.); ceegerlach@googlemail.com (C.E.G.); 2 Department of Physiotherapy, Chair of Physiotherapy, School of Health Sciences, Medical University of Silesia, 40-007 Katowice, Poland; aniabrzek@interia.pl; 3 Department of Cardiology I—Coronary and Peripheral Vascular Disease, Heart Failure Medicine, University Hospital Muenster, Cardiol, 48149 Muenster, Germany

**Keywords:** physical activity, diabetes mellitus, cognitive function, walking, E-health, exercise

## Abstract

Background: Lifestyle habits strongly influence health. It is strongly believed that physical activity may improve cognitive function. We examined the association between two kinds of physical activity and cognitive function in patients with type 2 diabetes. Methods: Using a random allocation sequence, 49 patients with type 2 diabetes (metformin, insulin, and diet-controlled) were randomized to a 12-week intervention of either walking 40 min three times a week (*n* = 17), performing pedometer-controlled activity (E-health, goal 10,000 steps a day, *n* = 17), or receiving standard care (*n* = 16 controls). We prospectively examined cognitive function, metabolic parameters, height, and weight. The groups were compared using linear regression adjusted for age. Results: Compared with the control group (*n* = 16), nonverbal memory improved significantly after the intervention in the walking group (*n* = 16) (28.2 (+/−6.1) vs. 35.3 (+/−5.3) *p* < 0.001) and the E-health (pedometer) group ((*n* = 17) (29.7 (+/−3.9) vs. 35.6 (+/−3.8) *p* < 0.001). The verbal memory test showed improvement in the walking and E-health groups. Cognitive attention/performance measured by the FAIR-test was also significantly enhanced in the walking group (252.4/304.3 *p* < 0.001, 51.87 (CI 27.13–76.62)) and the E-health-group (85.65 (CI: 52.04–119.26, *p* < 0.001)). Abdominal circumference (−3 cm (CI: −9.69–3.31, *p* < 0.001)), heart rate (−6.50 (CI: −9.69, −3.31, *p* < 0.001)) and fat percentage (−2.74 (CI: −4.71, −0.76, *p* < 0.007)) changed significantly in only the walking group. Conclusions: This is the first intervention study in patients with type 2 diabetes that shows that pedometer-supported training significantly improves brain function. Walking additionally improves body composition and waist circumference. Physical activity is an inexpensive treatment with substantial preventative and restorative properties for cognitive and memory brain function in patients with type 2 diabetes.

## 1. Introduction

Previous studies [1,2] report the positive influence of physical activity on cognitive function in old age patients or patients with diabetes. However, the differential effects of physical activity on cognitive function remain to be studied, and the presented study intended to prospectively test the influence of two different physical activities on cognitive function in patients with type 2 diabetes.

Physical activity is one of the most important therapy tools in terms of internal medicine [3]. Thirty minutes of physical activity daily can reduce mortality up to 31% [4]. People who are physically active are less likely to be depressed [5] or obese and have a lower risk of developing colon cancer [6]. Diabetes mellitus is one of the most significant challenges of our time [7,8]. In the upcoming years, almost a billion people will suffer from diabetes mellitus and metabolic syndrome [8,9,10]. One of the many causes of obesity, diabetes, and metabolic syndrome is a lack of exercise [11,12,13,14]. Other causes are excessive sugar intake [15] and social circumstances [16]. Diabetes mellitus destroys neuroplasticity and neuronal architecture, resulting in severely impaired cognition [17,18] and brain function [19,20]. Exercise benefits mental health [21]. Physical activity positively influences brain function [22,23] and neurogenesis [24,25]. “Run regular, age slower, be neuroplastic” was the slogan created by Dr. Khan [26]. Generally, physical activities contribute both to build diabetes type 2 clinical pathways in primary care [27,28] and to reduce the burden of chronic diseases, improving public health [28]. To the best of our knowledge, only one prospective and randomized trial about physical activity and cognition in patients with diabetes has been conducted and was performed from 2014–2016 in sedentary older patients [2]. Participants who could walk 400 m in 15 min were asked to walk three to four times a week for 30 min at a moderate intensity [2]. Patients with diabetes showed a greater improvement in mental function in the LIFE trial than patients without diabetes. The quote “exercise is brain food” indicates that physical activity leads to a significant improvement in cognitive function [29].

## 2. Methods

Type 2 diabetic patients (26 men/23 women, stably controlled with diet and/or metformin or insulin for at least six months) were randomized to the walking group (*n* = 16), the E-health group (pedometer, *n* = 17), or the control group (*n* = 16).

Participant characteristics are shown in Table 1. Participants were excluded if they suffered from any cardiac conditions, were regularly physically active (≥60 min moderate-vigorous activity per week), or had any contraindications to exercise stress testing according to the guidelines [30] or a trial conducted by Cassidy et al. [31].

The institutional review board of University Witten-Herdecke (184/2015) approved the trial. Informed consent was obtained from all participants. Participation was voluntary, and participants were free to withdraw from the study at any time without negative consequences. Data collection was performed in accordance with the guidelines set by the Declaration of Helsinki. We recruited participants by advertising through local newspapers, social media, contacts in practices, and diabetes community groups between October 2016 and January 2017.

Spiroergometry [32,33] was performed in the following manner: after successful gas and volume calibration, we completed the stress test beginning at 50 watts, continuously increasing by 25 watts every 2 min (ramp test). The test was accomplished when the participant could not maintain the predefined cadence of 80/min or if the participant was subjectively exhausted and there was no further increase in VO2 max after 20 s. Spiroergometric analyses were conducted as previously described [32,34]. The ventilator aerobic threshold (VAT) was defined as the first nonlinear increase in VCO2 output in relation to oxygen uptake [35]. The respiratory compensation point (RCP) was defined as the simultaneous nonlinear increase in both ventilatory equivalents and the crossing of CO2 output above the VO2 intake according to the previously described recommendations [35]. Body weight and body composition were determined using a Tanita BC-418MA segmental body composition analyzer [36]. The participants were instructed to wear only comfortable shorts during this test.

Blood was collected via venipuncture and analyzed (MVZ Dr. Stein + Kollegen, Mönchengladbach). Serum triglycerides, total cholesterol, and high-density lipoprotein (HDL) cholesterol were measured by enzymatic methods; fasting glucose was measured via the hexokinase method. Low-density lipoprotein (LDL) cholesterol was determined using the Friedewald equation. Height (stadiometer) and weight (digital scales) were measured without shoes to the nearest 0.5 cm and 0.1 kg, respectively. Blood pressure was measured three times in the seated position after ≥5 min of rest using an automated blood pressure monitor (Boso).

### 2.1. Experimental Protocol and Randomization

After initial screening, spiroergometry, body composition, and blood variables were measured at baseline and after 12 weeks of the walking intervention (40 min three times a week), pedometer-supported physical activity control (goal of 10,000 steps a day), or continued standard care. A fourth group was created (HIIT training), but some participants did not accept this training, and at the end, there were only six participants in this group, so they were not evaluated.

Participants were randomized into groups using a simple random list (performed by our statistical support (P-Point)). Concealed envelopes with consecutive numbers were locked in a drawer and withdrawn in numerical order by the primary author.

**Figure 1:**
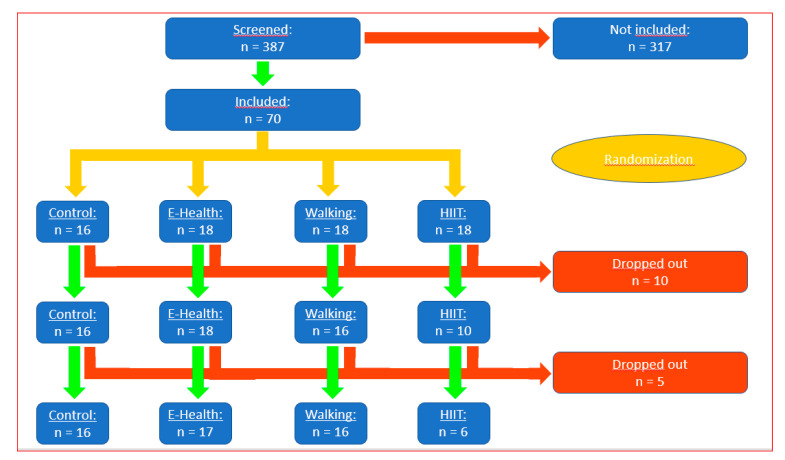
Flow diagram showing patients numbers at each stage of the trial.

A total of 387 patients were screened for the possibility of participating; 317 did not fulfil the criteria, and 70 were randomized. Sixteen patients were assigned to the control group and 18 to each of the intervention groups. Ten patients withdrew for unrelated or medical reasons after randomization in the other group, and five had to be excluded for technical reasons. In the E-health group, 18 patients started, one dropped out. Finally, the data of 17 patients from the E-health group were evaluated. 18 patients were randomized into the walking group, 16 started with walking, and all 16 were evaluated. All 16 randomized patients to the control group were evaluated. Eighteen patients were randomized to the high-intensity interval training (HIIT) group, ten (10) started with the study, but only 6 finalized the course of 12 weeks. The HIIT group had to be dissolved because too many patients dropped out (Figure 1).

### 2.2. Measurement of Cognitive Function

We used the FAIR-Test 2 to measure attention [37]. The revised version of the FAIR-Test 2 has been available since 2011. This test provides an error-corrected ***performance value P*** (referred to as the working pace). Additionally, FAIR 2 provides a ***quality value Q*** (a measure of caring and relative accuracy in the processing of the task = proportion of the concentrated judgements out of all judgements) and ***continuity value K*** (calculated as the product of L and Q; as the extent of the continuously given concentration).

### 2.3. Verbal (VM) and Nonverbal Memory (NVM)

Determining the verbal memory (VM), the test participants initially read aloud simple sentences and later short stories. The text should be reproduced with the same words. The number of correctly reproduced words was determined, where only the same terms were counted, and no nouns replaced by personal pronouns were evaluated [38].

The nonverbal memory capacity (nV) was determined by showing the participants pictures that should be recognized. The images consisted of simple objects but also abstract constructions. For this purpose, the participant was shown a picture for five seconds. Then, the picture should be recognized among several different pictures. This should be done within 20 s. If the participant failed in the first attempt, another 10 s were given for a second try. Each picture recognized in the first attempt received two points; for correctly identified images in the second attempt, one point was awarded, otherwise, no points were given. If the participant achieved zero points twice in a row, the test was ended, and the points were added. The maximum number of points that could be achieved was 92. The pictures shown were age-dependent. The test could be evaluated if two tasks were answered correctly at the beginning. The points achieved were added up at the end. A maximum of 113 points could be obtained [37,38,39].

## 3. Results

All participants in the walking group completed the intervention with 40 min walking 3x/week. In the E-health group, 88.24% of the 12 weeks steps targets were completed. The mean value of the reached steps in the e-health group was 8774/day (standard deviation 3995/day).

Participant processing and the number of randomized participants are shown in Figure 1. Finally, we evaluated 16 participants in the control group, 16 participants in the walking group, and 17 participants in the E-health (pedometer) group. The groups were well matched for all baseline characteristics (Table 1). The differences from pre- to post-intervention are listed in Table 2.

**Figure 2:**
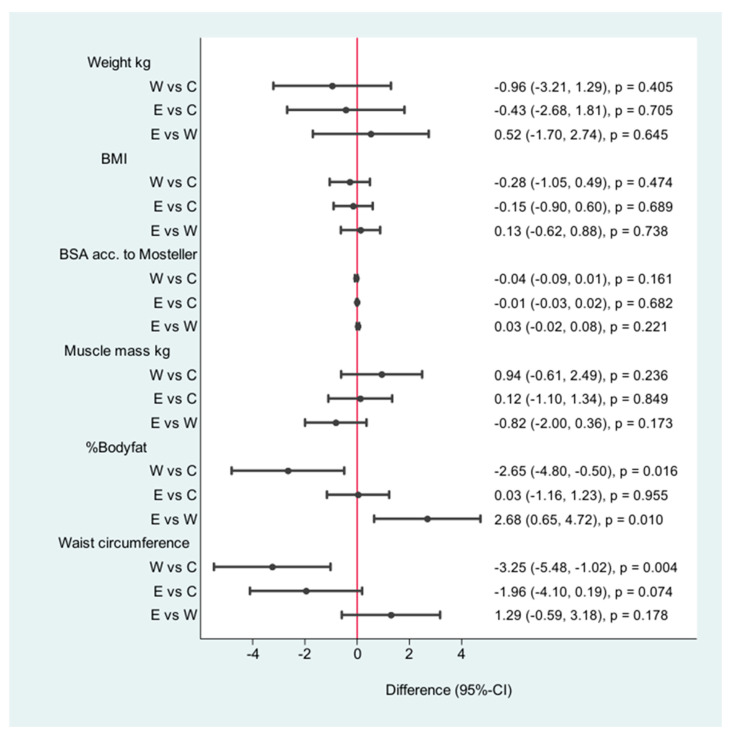
Differences after the intervention W = walking, C = control, E = E-health.

**Figure 3:**
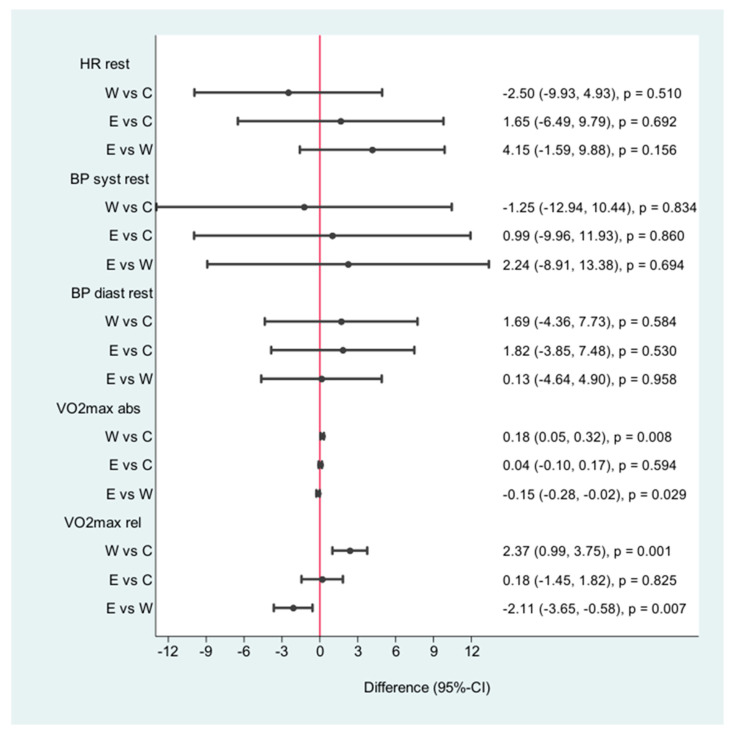
Differences after the intervention; HR = heart rate, BP = blood pressure, VO2 max abs = oxygen uptake max, VO2 max rel = oxygen uptake in relation to weight. W = walking, C = control, E = E-health (pedometer).

The walking group was more successful in reducing the waist circumference (−3 cm, CI: −4.41–1.59) *p* < 0.001) and in reducing body fat (−2.74%/CI: −4.71, −0.76/*p* < 0.007) than the control group and the pedometer group (Figure 2). The heart rate at rest changed significantly (6.50 *p*/min/CI: −9.69, −3.3/<0.001). The pedometer group was more effective in reducing waist circumference than the control group (−1, 71 cm, CI: 3.00–041, *p* < 0.010) (Figure 3).

Cognitive performance improved significantly (participants thought faster) in both physically active groups (walking 51.87 (27.13, 76.62) (*p* < 0.001) and the E-health group (85.65 (52.04, 119.26) (*p* < 0.001)). We summarized the results in Table 2.

### 3.1. Weight/Body Composition

In the standard care group, no significant changes were observed in any parameters (Table 2, Figure 2). In the walking group, there was no marked tendency to change in weight (according to the improvement in muscle mass). The mean difference was a nonsignificant weight loss of 1.17 kg. However, body fat reduction in the walking group was 7.1% in comparison with that at baseline. The E-health group showed no significant difference in weight after the intervention (Figure 2).

### 3.2. Spiroergometry

Relative oxygen intake (VO2 max in mL/min/kg) changed in a positive direction only in the walking group (0.91 CI: −0.03, 1.85, *p* < 0.058). We observed a reduction in worsening over the 12 weeks in the control group and the same in the pedometer group. Achieving the step goal did not lead to measurable improvement in oxygen intake within 12 weeks. The results are shown in Table 2 and Figure 3.

### 3.3. Cognition

**Figure 4:**
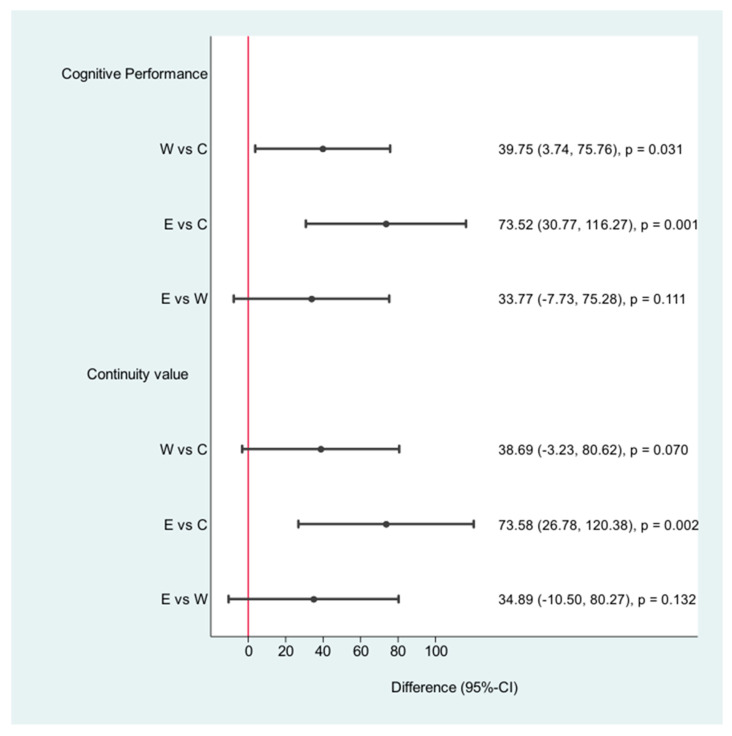
Cognition: differences after the intervention; W = walking, C = control, E = E-health.

**Figure 5:**
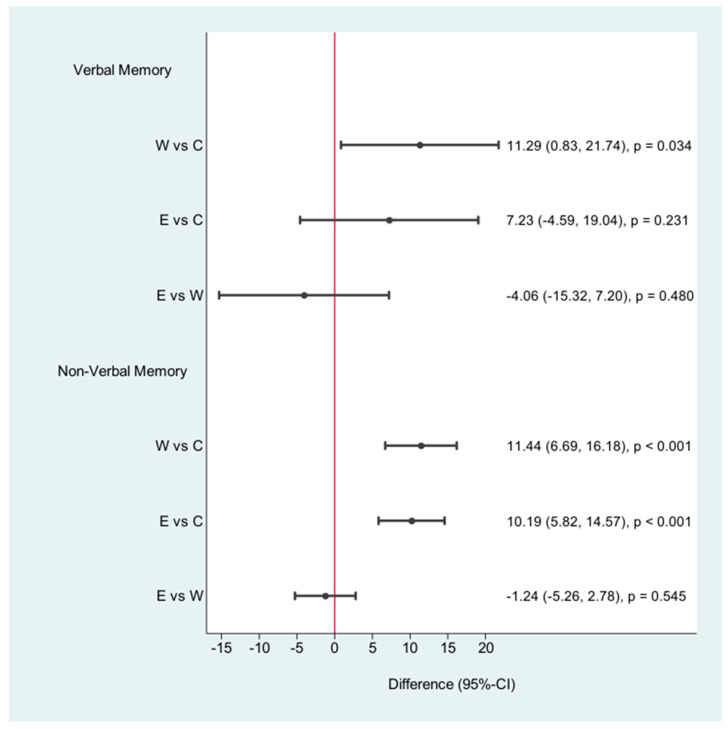
Verbal and nonverbal memory: differences after the intervention; W = walking, C = control, E = E-health.

Cognitive performance improved in both groups after the intervention (Table 2, Figure 4 and Figure 5). Walking and counting steps led to enhanced cognitive performance pace and concentration. The standard care group was only asked to walk 30 min per day. This statement alone does not lead to any changes. Values for VM improved significantly for both intervention groups. The walking group exhibited exceptionally improved VM values (12.00 CI: 4.79, 19.21, *p* < 0.001) and nonverbal memory values (7.13 (3.96, 10.29, *p* < 0.001). The pedometer group showed a mental benefit in cognitive performance (85.65 (52.04, 119.26) *p* < 0.001) and nonverbal memory values (5.88 (3.26, 8.50) *p* < 0.001). VM showed no significant improvement. The walking group showed the best results.

## 4. Discussion

“Exercise makes you feel good” is a common assumption that refers to single or repeated bouts of physical activity [40]. After our study, we can add the following: physical activity improves cognitive function in patients with diabetes. The present research is the first prospective randomized trial to examine the effects of a pedometer intervention (compared to a walking intervention) on cognitive functions in patients with type 2 diabetes. Today, diabetes has become a very large public health burden [41]. The decline in cognitive function with age is an additional problem. Currently, there are approximately 1.4 billion people over 55 years worldwide. The objective deterioration in cognitive performance accelerates around the age of 50 years [22]. Physical activity enhances cognitive performance in older people without cognitive impairment [22]. In 2003, Colcombe et al. [42] described in a meta-analysis that aerobic training improves cognitive function in older healthy people. Prospective intervention studies referring to cognitive function and diabetes are rare. Only one prospective study [43] showed improved cognitive function in older patients with diabetes but not in people without diabetes. This intervention included walking, with a goal of 150 min/week; strength; flexibility; and balance training [43]. A bias in the LIVE trial was that the participants with diabetes were younger than those in the control group [2]. In our research, there were no significant differences between the study groups. We showed that physical activity, such as counting steps or walking a minimum number of kilometers per week, improved nonverbal memory and cognitive performance. Walking three times a week seems to be more effective in decreasing waist circumference and reducing body fat. Nevertheless, step counting can help maintain cognitive function in a similar way as walking three times a week. In older people with mild cognitive impairment who are at risk for Alzheimer’s disease, physical activity interventions lead to a modest improvement in cognitive function [1]. On the other hand, Eggermont et al. [44] described in a randomized trial a lack of beneficial effects of walking programs on cognition in older nursing home residents with moderate dementia. The authors discuss that the effect of walking (5 × 30 min, 6 weeks) in patients with dementia was reduced because the level of activity was low (due to cardiovascular comorbidity), which hindered any significant improvement in cognition.

The influence of physical activity on depression is a widely studied area. It is possible that physical activity improves depression, but a meta-analysis of 14 studies did not find any effect of physical activity on depression [45].

These findings have not been confirmed, so the statement “physical activity improves cognition” cannot definitively be considered false. Clinical or subjective observations are one component of research, and prospective clinical trials are another; patient observations differ from the results of prospective clinical trials.

In many trials with participants with dementia or cognitive impairment, the level of the preexisting disorders was not clearly defined. That is why trials on improving cognitive function through physical activity more often show a beneficial effect in healthy people, but the results cannot be transferred to mentally ill individuals. A randomized trial in older healthy women (70–93 years) revealed that an exercise routine could significantly improve cognitive function over six months [46]. These women were mentally healthy, and exercise interventions work in healthy people very well. The same positive influence of aerobic exercise was described by Barcelos et al. [47]. Riding a stationary bike improves cognition in older adults, and additional computer gaming further enhances the results. Loprinzi et al. [48] described evidence that acute, short-duration exercise may help attenuate a retroactive memory interference effect. Implications of these findings in regard to using exercise to improve memory and attenuate memory decay are discussed.

Patients in our study were mentally healthy, and their main disorder was diabetes with all related conditions (metabolic syndrome, microangiopathy, etc.). After our study, we can state that achieving a daily minimum or mean value of 8700 steps or walking three times 40 min per week can improve cognition in patients with type 2 diabetes. Our research is the first trial with participants suffering from type 2 diabetes who showed an improvement in cognition as a result of an intervention with physical activity in a study population without differences at baseline. The influence of physical activity or fitness on cognition is not evident. Erickson et al. [49] comment in a review paper that there is now substantial evidence that higher fitness levels are connected with better cognitive health among children and older adults. The problem is to define the duration and level of training that will improve cognitive function. The question regarding how much exercise is enough remains widely discussed [50]. For physical fitness, 10,000 steps is one goal, but the definition of “volume” or intensity of physical activity necessary to improve cognition is not 100% clear. This study includes a small groups of individuals, but from the statistical point of view we reached significant differences for an interventional study (more individuals than in the study from Cassidy et al. [31]. For the future, we have to test our results in a greater collective.

In conclusion, from our trial, we can state that a minimum of ca. 8700 steps daily (the mean value of our participants) should be achieved to improve cognitive function. In our opinion, a particular walking intervention should be preferred because its additional effects on body composition and waist circumference are evident. Using a pedometer is better than nothing. For future options to treat patients with diabetes, we have to improve our infrastructure and create walking possibilities to prevent sedentary behavior [51,52]. Regarding the treatment of diabetes beyond skeletal muscle [53] and metabolic health [54], we should be aware of the option to improve cognitive function in individuals with diabetes through physical activity. In recent studies [53,54,55], the beneficial effect of exercise on cognitive function has tended to be neglected. The cognitive impairment/improvement mechanisms in patients with diabetes are complex [56,57], and further studies on this issue are needed. Walking a minimum distance or counting steps (through the use of a pedometer device) are inexpensive therapeutic options for improving brain function, which declines in the early stage of diabetes mellitus disease [58].

## Figures and Tables

**Table 1:** Baseline characteristics and comparison of the three examined groups. BMI: Body Mass Index, BSA: Body Surface Area, HR: Heart Rate, BP: Blood Pressure, VO2 max abs: Oxygen uptake absolut, VO2 max rel: Oxygen uptake in kg/mL/min.

	Control (*n* = 16) Mean (sd)	Walking (*n* = 16) Mean (sd)	E-Health (*n* = 17) Mean (sd)	*p*-Value *
Age, years	59.1 (8.5)	60.4 (5.9)	56.4 (8.8)	0.310
Weight, kg	98.6 (16.5)	96.7 (15.4)	103.7 (18.0)	0.478
BMI	33.8 (4.4)	34.4 (4.6)	33.8 (5.2)	0.924
BSA acc. to Mosteller	2.16 (0.23)	2.12 (0.21)	2.24 (0.22)	0.259
Muscle mass, kg	60.7 (12.9)	55.4 (11.3)	63.8 (10.0)	0.092
%Bodyfat	35.5 (7.5)	39.5 (8.7)	34.6 (7.0)	0.194
Waist circumference	114.1 (11.6)	114.2 (10.4)	116.3 (14.5)	0.868
HR rest	77.6 (11.3)	76.4 (10.7)	75.3 (9.3)	0.819
BP syst rest	137.9 (12.6)	140.6 (11.7)	135.7 (12.6)	0.521
BP diast rest	84.4 (6.3)	83.0 (8.6)	81.2 (7.6)	0.426
VO2 max abs	1.78 (0.67) (*n* = 10)	1.59 (0.39) (*n* = 14)	1.97 (0.43) (*n* = 14)	0.067
VO2 max rel	18.1 (5.6) (*n* = 10)	16.4 (3.4) (*n* = 14)	19.9 (3.5) (*n* = 14)	0.039
Haemoglobin g/dL	14.2 (1.3)	14.9 (1.2)	14.6 (1.3)	0.266
HbA1c %	7.0 (1.3)	7.3 (1.3)	6.9 (0.8)	0.467
HbA1c mmol/mol	52.8 (14.5)	56.6 (14.0)	51.2 (8.4)	0.420
Blood sugar	154.9 (41.9)	156.9 (39.8)	144.1 (29.0)	0.499
Cholesterol mg/dL	186.0 (29.1)	199.0 (40.0)	193.3 (43.6)	0.563
Triglycerides mg/dL	199.9 (64.9)	185.7 (114.5)	237.6 (173.5)	0.596
Cholesterol HDL mg/dL	50.2 (10.8)	52.4 (13.3)	45.9 (12.5)	0.342
Cholesterol LDL mg/dL	112.6 (33.8)	121.2 (32.7)	113.6 (35.2)	0.738
Cognitive performance	240.1 (35.6)	252.4 (66.8)	266.6 (80.8)	0.435
Quality value (%)	89.8 (13.2)	91.0 (10.0)	88.6 (9.3)	0.777
Continuity value	230.1 (63.2)	228.2 (61.6)	243.4 (83.2)	0.824
Verbal memory	60.0 (19.9)	66.4 (11.3)	62.9 (15.9)	0.510
Nonverbal memory	32.4 (4.9)	28.2 (5.1)	29.7 (3.9)	0.061

* F-Test. HDL: High Density Lipoprotein, LDL: Low Density Lipoprotein.

**Table 2:** Calculated differences and confidence interval. *p* * sigificant *p* < 0.05.

	Control		Walking		E-Health	
	Diff. (95%-CI)	*p* *	Diff. (95%-CI)	*p* *	Diff. (95%-CI)	*p* *
Weight kg	−0.21 (−1.85, 1.43)	0.799	−1.17 (−2.76, 0.43)	0.151	−0.65 (−2.24, 0.94)	0.425
BMI	−0.09 (−0.64, 0.46)	0.747	−0.37 (−0.93, 0.19)	0.191	−0.24 (−0.77, 0.28)	0.364
BSA acc. to Mosteller	−0.00 (−0.02, 0.02)	0.835	−0.04 (−0.08, 0.01)	0.121	−0.01 (−0.02, 0.01)	0.439
Muscle mass kg	−0.12 (−1.26, 1.02)	0.838	0.82 (−0.27, 1.91)	0.140	0.00 (−0.55, 0.55)	1.000
%Bodyfat	−0.09 (−1.04, 0.86)	0.857	−2.74 (−4.71, −0.76)	0.007	−0.05 (−0.81, 0.71)	0.892
Waist circumference	0.25 (−1.53, 2.03)	0.783	−3.00 (−4.41, −1.59)	<0.001	−1.71 (−3.00, −0.41)	0.010
HR rest	−4.00 (−10.85, 2.85)	0.252	−6.50 (−9.69, −3.31)	<0.001	−2.35 (−7.14, 2.43)	0.335
BP syst rest	−6.75 (−15.00, 1.50)	0.109	−8.00 (−16.55, 0.55)	0.067	−5.76 (−13.25, 1.72)	0.131
BP diast rest	−4.88 (−9.78, 0.03)	0.051	−3.19 (−6.89, 0.52)	0.092	−3.06 (−6.21, 0.09)	0.057
VO2max abs	−0.12 (−0.22, −0.03)	0.014	0.06 (−0.04, 0.15)	0.224	−0.09 (−0.18, 0.00)	0.061
VO2max rel	−1.47 (−2.50, −0.45)	0.005	0.91 (−0.03, 1.85)	0.058	−1.18 (−2.33, −0.03)	0.044
Hemoglobin g/dL	−0.02 (−0.49, 0.45)	0.937	−0.15 (−0.47, 0.17)	0.355	0.18 (−0.17, 0.53)	0.324
HbA1c %	0.04 (−0.48, 0.56)	0.888	−0.03 (−0.34, 0.29)	0.877	−0.19 (−0.47, 0.09)	0.192
HbA1C mmol/mol	0.04 (−5.64, 5.71)	0.990	−0.38 (−3.71, 2.96)	0.825	−1.84 (−4.93, 1.26)	0.245
Blood sugar	−1.50 (−16.80, 13.80)	0.848	−2.56 (−22.10, 16.97)	0.797	−3.12 (−18.93, 12.69)	0.699
Cholesterol mg/dL	−4.13 (−12.48, 4.23)	0.333	−14.25 (−28.20, −0.30)	0.045	−8.65 (−18.55, 1.25)	0.087
Triglycerides mg/dL	−27.00 (−58.57, 4.57)	0.094	3.06 (−27.81, 33.94)	0.846	−27.06 (−71.44, 17.32)	0.232
Cholesterol HDL mg/dL	−0.06 (−2.94, 2.81)	0.966	0.63 (−3.36, 4.61)	0.759	0.94 (−1.29, 3.17)	0.408
Cholesterol LDL mg/dL	−11.81 (−22.65, −0.98)	0.033	−23.18 (−35.30, −11.05)	<0.001	−12.81 (−21.19, −4.44)	0.003
Cognitive Performance	12.12 (−14.85, 39.10)	0.378	51.87 (27.13, 76.62)	<0.001	85.65 (52.04, 119.26)	<0.001
Quality value (%)	−1.13 (−9.17, 6.92)	0.784	3.52 (−1.52, 8.56)	0.171	3.88 (0.85, 6.91))	0.012
Continuity value	16.35 (−14.95, 47.66)	0.306	55.05 (26.12, 83.97)	<0.001	89.94 (54.45, 125.42)	<0.001
Verbal Memory	0.71 (−7.12, 8.55)	0.858	12.00 (4.79, 19.21)	<0.001	7.94 (−0.84, 16.72)	0.076
Non-Verbal Memory	−4.31 (−7.95, −0.67)	0.020	7.13 (3.96, 10.29)	<0.001	5.88 (3.26, 8.50)	<0.001

## Data Availability

The datasets analyzed during the current study are available from the corresponding author upon reasonable request.
